# Unanswered Questions in HIV Hematology

**DOI:** 10.1155/2012/907025

**Published:** 2012-06-04

**Authors:** Heather A. Leitch, Jeremy S. Abramson, Matthew C. Cheung, Lisa K. Hicks

**Affiliations:** ^1^Division of Hematology, St. Paul's Hospital and the University of British Columbia, 440-1144 Burrard Street, Vancouver, BC, Canada V6Z 2A5; ^2^Centre for Lymphoma, Massachusetts General Hospital Cancer Centre and Harvard Medical School, Boston, MA 02114, USA; ^3^Division of Hematology, Sunnybrook Health Sciences Centre, University of Toronto, Toronto, ON, Canada M4N 3M5; ^4^Division of Hematology, St. Michael's Hospital, University of Toronto, Toronto, ON, Canada M5S 1A1

Hematological abnormalities are common manifestations of HIV infection that are regularly encountered in the clinical setting. Although initially recognized with the first descriptions of HIV infection over 30 years ago, our understanding of the epidemiology, natural history, and treatment of these blood disorders continues to evolve. Aggressive lymphomas such as diffuse large B-cell lymphoma and primary CNS lymphoma have decreased since the introduction of combination antiretroviral therapy (cART), though these and other HIV-associated malignancies continue to a have a major impact on the morbidity and mortality of persons with HIV. Other blood disorders frequently associated with HIV include cytopenias, particularly anemia and thrombocytopenia. In this special issue, studies investigating hematologic diseases associated with HIV infection are discussed. The diagnosis, treatment, and prognosis of Hodgkin lymphoma (HL) in the era of cART are reviewed by Jacobson and Abramson. Unlike non-Hodgkin lymphomas (NHL), the incidence of HL in HIV-infected patients has not decreased in the cART era. However, the introduction of cART has allowed the delivery of full doses of chemotherapy with significantly improved outcomes. Despite these advances, patients with HIV-associated HL remain at risk of treatment-related toxicity, and interactions between antiretroviral and chemotherapeutic agents necessitate careful attention to supportive care.

Two papers focus on HIV-associated Burkitt lymphoma (BL). Though the outcome of HIV-NHL has improved substantially in the cART era, the outcome of HIV-BL with standard chemotherapy remains poor. J. A. Rodrigo et al. describe the combined experience in four Canadian centers treating patients with HIV-BL with the CODOX-M/IVAC regimen with or without rituximab in the modern era. In this study, intensive chemotherapy with CODOX-M/IVAC ± R yielded acceptable toxicity and favorable survival rates. A. M. Petrich et al. review the larger picture in the treatment of HIV-BL in the paper entitled “*paradigms and controversies in the treatment of HIV-related Burkitt lymphoma*.” In this paper, available data on the treatment of patients with HIV-BL with current BL-directed chemotherapy regimens are discussed in detail, including Hyper-CVAD, dose-adjusted EPOCH, the PETHEMA regimen, and CODOX-M/IVAC. Areas of uncertainty in the treatment of these patients include the addition of rituximab to chemotherapy regimens, the optimal approach to patients with relapsed or refractory BL and the role of stem cell transplantation, the appropriate approach to older patients and patients with central nervous system involvement, and the role of antiretroviral therapy and supportive care.

An intriguing and pressing challenge in the management of patients with both malignant and nonmalignant hematologic disorders in HIV infection is the optimal approach to be taken in the developing world. This topic is addressed by M. Ulrickson et al. in the paper “*Epidemiology, diagnosis, and treatment of HIV-associated NHL in resource-limited settings.*” The epidemiology of NHL in Africa is compared to that of the US, and discrepancies in survival highlighted by cancer registries are discussed. Challenges in managing patients with NHL in resource-limited settings include diagnostic challenges due to limited access to a full battery of immunohistochemical tests and limited molecular testing. Treatment challenges include limited access to antiretroviral regimens, prophylactic agents, IV infusion centers, and a higher incidence of major infectious complications including tuberculosis and hepatitis B. Response assessments are further limited by a lack of ready access to cross-sectional imaging in some regions. Despite these challenges, progress has been made in developing tolerable and effective treatment regimens appropriate to these circumstances, and these important advances are reviewed.

A common nonmalignant complication of HIV infection is immune thrombocytopenia (ITP). The characteristics of HIV-associated ITP were documented prior to the cART era, and the optimal treatment beyond cART is unknown. For this reason, K. L. S. Ambler et al. present a retrospective cohort study reviewing individuals with severe HIV-associated ITP diagnosed in the cART era. Their series is the largest such report of severe HIV-ITP in the post-cART era. The major finding from this study was that, although the various treatments were well tolerated and most patients achieved a safe platelet count, nearly all patients (87%) required retreatment for recurrence of severe ITP. This highlights that new approaches to the treatment of ITP in this population are needed.

The intent of this special issue was to initiate interest in and further inquiry into the many hematologic complications faced by persons living with HIV. In addition to the topics discussed in this issue, areas for future exploration include the approach to management of patients with less common lymphoproliferative disorders such as indolent NHL, paraproteinemias, and Castleman disease, as well as non-malignant causes of cytopenias and their management.

*Heather A. Leitch* *Jeremy S. Abramson* *Matthew C. Cheung* *Lisa K. Hicks*

